# Advantages and Disadvantages of Using St. John's Wort as a Treatment for Depression

**DOI:** 10.7759/cureus.29468

**Published:** 2022-09-22

**Authors:** Johanna S Canenguez Benitez, Tabata E Hernandez, Ramaswamy Sundararajan, Sarosh Sarwar, Angel J Arriaga, Asma T Khan, Andrea Matayoshi, Herbert A Quintanilla, Hansini Kochhar, Mehwish Alam, Arpit Mago, Aakash Hans, Guadalupe A Benitez

**Affiliations:** 1 Internal Medicine, Larkin Community Hospital, South Miami, USA; 2 Internal Medicine, Jagadguru Sri Shivarathreeshwara (JSS) Academy of Higher Education and Research, Bangalore, IND; 3 Medicine and Surgery, Fazaia Medical College, Islamabad, PAK; 4 Neurology, Sylhet MAG Osmani Medical College, Sylhet, BGD; 5 Medicine, Universidad Peruana de Ciencias Aplicadas, Lima, PER; 6 Psychiatry and Behavioral Sciences, Larkin Community Hospital, South Miami, USA; 7 Clinical and Translational Research, Larkin Community Hospital, South Miami, USA; 8 Psychiatry and Behavioral Sciences, Avalon University School of Medicine, Kenner, USA; 9 Internal Medicine, Jawaharlal Nehru Medical College, Belgaum, IND; 10 Research, Henry Ford Health System, Detroit, USA; 11 Research, Larkin Community Hospital, South Miami, USA

**Keywords:** moderate depression treatment, mild depression treatment, management of depression, menopause management, treatment for depression, st. john’s wort

## Abstract

Background and objectives: St. John's wort (SJW) extracts are currently being used to treat depression of various degrees of severity. While many studies have shown it to be superior to placebo, data regarding the effectiveness of using SJW as a stand-alone treatment compared with standard antidepressants has yet to be proven conclusively. This study aims to understand the advantages and disadvantages of SJW as a treatment modality for depression.

Methods: The authors searched PubMed, JAMA network, Springer Link, Elsevier, Google Scholar, and Scientific Progress databases, from 2011 through August 2021, using the following keywords: St John’s wort, Hypericum perforatum, depression, antidepressant, complementary alternative medicine, economic evaluation depression St. wort, St John’s wort and depression, antidepressant interactions. This yielded a total of 27 papers following a thorough removal of irrelevant content and dissemination in languages other than English.

Results: In patients with mild and moderate depression, SJW proved superior to placebo. Certain studies comparing the efficacy of SJW versus selective serotonin reuptake inhibitors (SSRIs), especially fluoxetine, reported SJW to be more efficacious, while the majority reported no significant difference. Tricyclic antidepressants were also found to have similar efficacy as SJW. Moreover, treatment with SJW was also found to reduce postmenopausal depression. Regarding the safety profile, although SJW is better tolerated with fewer adverse effects when compared to standardized antidepressants, its predisposition to causing fatal serotonin syndrome, when used in conjunction with other serotonergic agents and drug interactions noted with CYP 450 drugs, raises a question in the safety profile.

Conclusion: It is essential to acknowledge that SJW has been used as a treatment measure in Germany. Despite being only listed as a dietary supplement by the FDA and not a drug, SJW has shown to be comparable, if not more efficacious, than most standard treatment options for depression. SJW does prove to be an exciting piece of pharmacotherapy in the realm of mental health and post-menopausal treatment. More prospective studies will help us better understand its efficacy in mild and moderate depression and its ability to serve as a long-term agent. Considering its mechanism of action, its role in relieving patients suffering from an anxiety disorder is also worth considering.

## Introduction and background

Over the last few years, research about mental health has become increasingly important worldwide since the prevalence of mental disorders has increased enormously, with depression being the most common disorder. According to the World Health Organization, by 2017, 322 million people were suffering from this disorder worldwide [1]. In the United States (US) alone, there were 17.3 million adults who had suffered from major depressive episodes [2]. Unfortunately, the following years have been challenging. With the COVID-19 pandemic lockdown and millions of lost lives, many mental disorders have been left unattended and new cases of depression have emerged. A cross-sectional study compared the prevalence of depression symptoms in the US population before and after the COVID-19 pandemic; researchers found a three-fold increase [3]. A variety of medical treatments are well-known and available for patients. However, not all patients seek medical attention or can afford those prescribed medications. Therefore, they seek other types of options, such as traditional medicine.

In European countries, people have been using St. John's wort (SJW) as an essential part of plant-based medicine practices. SJW is a yellow flower, and its name goes far back to the ancient Greeks; it refers to John the Baptist, as the plant blooms around late June, which is the time of the feast of St. John the Baptist. Generally, it was mainly used for the treatment of a variety of mental conditions such as insomnia, depression, attention-deficit hyperactivity disorder (ADHD), somatic symptom disorder, generalized anxiety disorders, obsessive-compulsive disorder, and other diseases [4].

Even though many uses have been related to this plant; there is extensive research that supports the use of SJW for depression, as it appeared to be more effective than a placebo (an inactive substance) and can be considered as effective as standard antidepressant medication for mild and moderate depression [5]. At the same time, it has been clearly shown that SJW can interact dangerously with various other medicines. It is considered an activator of the cytochrome P450 metabolism in the liver, which can potentially interact to cause life-threatening conditions [5].

SJW comes from flowering tops of Hypericum perforatum that constitute flavonoid derivatives, anthracenes, xanthones, volatile oils, proanthocyanidins, tannins, and caffeine acid derivatives [6]. Lecrubier Y et al. conducted a double-blinded RCT study in 2002, in which significant improvement in depression was seen among the SJW-treated group compared to the control group [6,7]. However, another study on 200 depressed patients did not differ from the control group [8]. Linde et al. did a meta-analysis that showed marked superiority of SJW compared to the placebo among 23 randomized clinical trials among mild to moderate depressive patients. However, compared to other antidepressants, almost the same benefits were seen in both groups, except fewer side effects were seen in SJW [8,9]. Another systematic review and meta-analysis also showed the superiority of SJW extracts to placebo and had similar efficacy to some traditional antidepressants [10]. Consistent with these two studies, randomised controlled trials done in 2009 showed the same responder rate of hypericum extract such as paroxetine, but a better tolerance was seen in the hypericum group [11]. UK guidelines for depression in adults do not support the use of SJW due to a lack of necessary data regarding dosage, variation in nature of preparation, drug interactions (with oral contraceptive pills, anticoagulants, and anticonvulsants), and persistence of effect [12].

SJW's efficacy has been well established in moderate to severe depression [13]. However, despite significant evidence proving its use compared to other standard antidepressants, SJW's use in mild and moderate depression and its long-term efficacy are still unclear and need further assessment [14]. Another matter to address is drug interactions. SJW has been shown to interact and cause changes to the pharmacokinetics of some drugs such as digoxin, tacrolimus, warfarin, and alprazolam [15]. 

Addressing the issues listed above is imperative simply because it will give us a pellucid viewpoint on the use of SJW when combined with other standard antidepressants like sertraline and paroxetine. In addition, patient beliefs are also a key aspect to consider. Understanding the patient's mentality will help better prepare clinicians to decide between various options for the treatment of depression [16].

Due to the wide popularity of SJW and its role in depression, it is essential to have a firm hold and a good understanding of the topic. Furthermore, with an increasing number of patients using SJW for depression, our duty as medical professionals is to be updated about its effects on human physiology. Therefore, we aim to research the literature on the role of SJW in the diagnosis, treatment, and prognosis of depression of both mild and moderate severity. This shall involve a literature review of relevant articles which discuss SJW and depression. After a cursory glance, published articles will either be included or eliminated according to whether they meet the inclusion criteria. The selected articles would then be reviewed, and significant advances in SJW would be studied and further investigated in our review. 

Methods

This study selected 27 articles from Pubmed, JAMA Network, Google Scholar, Springer Link, Elsevier, Scientific Progress, and other databases and journals. The reviewed articles included systemic reviews, research articles, cross-sectional studies, randomized control trials, literature reviews, and clinical trials. This article aims to inform the audience about the significance of SJW in the diagnosis, treatment, and prognosis of mild and moderate depression. The keywords used were St John wort depression, economic evaluation depression St. Wort, complementary and alternative medicine depression, and Hypericum perforatum, depression.

The inclusion criteria included academic articles published in the last ten years and written in English. The exclusion criteria were articles in languages other than English as well as non-academic articles.

## Review

SJW's efficacy as combined therapy with other antidepressants

Despite the variety of research about SJW use for depressive disorders, there is not much literature about its adjuvant with other antidepressive treatments. One study in Iran compared the efficacy of tricyclic antidepressants versus those combined with SJW in 40 patients with major depressive disorder. The latter showed a mild improvement in treatment [6]. However, most systematic reviews do not mention the adjuvant, while comparisons between SJW and placebo or an antidepressant have been well studied. It is essential to note that most studies comparing SJW efficacy were done in patients with mild and moderate depression.

Selective serotonin reuptake inhibitors (SSRIs) are the first-line therapy for this type of disorder in most countries; hence, the comparison is mainly with this type of antidepressant, as shown in Table 1 [17]. Different systematic reviews agree that SJW shows better results in treating patients with mild/moderate depression compared to placebo; however, when compared to SSRI, there is no significant difference [17-19]. Only a subgroup analysis of a double-blind control trial showed that SJW was superior to the SSRI paroxetine in patients with moderate depression after six weeks of treatment [20]. However, it was seen that patients using SJW had fewer adverse side effects, gastrointestinal and neurologic, which was also related to the lower discontinuation or dropout rate compared to those with conventional antidepressants. 

**Table 1:** Differences between St. John's wort and the most common antidepressant groups SSRI: selective serotonin reuptake inhibitors; GABA: gamma-aminobutyric acid; NSAIDs: non-steroidal anti-inflammatory drugs.

PARAMETER	St. JOHNS WORT	TRICYCLIC ANTIDEPRESSANT (TCA)	SEROTONIN REUPTAKE INHIBITORS (SSRI)
Efficacy	Almost the same as conventional antidepressants	Effective treatment for depression	First-line treatment for depression
Safety	Same as conventional antidepressants	Safe use	Safe use
Most common adverse effect	Less than conventional antidepressants	Cardiac arrhythmia	Serotoninergic syndrome
Mechanism of action	Inhibition of the reuptake of serotonin monoamine oxidase activity reduces GABA binding.	Inhibition of the reuptake of serotonin and norepinephrine	Inhibition of the reuptake of serotonin
Cost	Low	Higher	High
Rate of discontinuation due to side effects	Low	Higher than St John's Wort	Higher than St John's Wort
Withdrawal symptoms rate	Low	Higher than St John's Wort	Higher than St John's Wort
Long-term antidepressant effects	Limited data	Well known long effectiveness	Well known long effectiveness
Approved by FDA	No	Yes	Yes
Drug interactions	HIV drugs, ciclosporin, tacrolimus, digoxin, oxycodone, warfarin, etc.	SSRI, anticholinergic, antihypertensive. antihistamine, etc.	NSAIDs, aspirin, warfarin, and all drugs increase serotonin, etc.

SJW's efficacy versus traditional depression management (SSRI and tricyclic antidepressants)

SSRIs have been used commonly to treat depression. Over the years, due to their collective side effect profile, SJW was studied to see if it possibly had any comparable efficacy. It is essential to state that before checking SJW's efficacy with that of SSRIs, their efficacy was compared to placebo. There was a remarkable improvement in its effectiveness in major depressive disorder instead of placebo [17]. Many studies compared SJW to fluoxetine. It has been shown that SJW seems to be more efficacious than fluoxetine in treating major depressive disorder [21]. It has also been shown through randomized controlled trials that SJW was comparable in efficacy to fluoxetine in mild to moderate depression [22,23]. It is important to note that studies show tricyclic antidepressants have similar efficacy to SJW. However, due to the significant side effects that tricyclic depressants cause, SJW has been touted as a possible safer option [24].

While efficacy is critical in deciding treatment options, safety profile plays a crucial role in patient compliance and long-term use. Studies have shown that SJW has a propensity to cause adverse effects to a lesser degree than SSRIs [25]. Sertraline seems to be associated with more side effects than SJW, especially two weeks after beginning treatment, according to van Gurp et al. [26]. Nausea and sexual difficulties were more with sertraline. However, adverse events such as headaches and sleep disturbance were comparable or seen more in patients who were started on SJW [27].

SJW treatment in postmenopausal depression

Menopause is the irreversible termination of menstruation after the female reproductive life ends, resulting in women having difficulties psychologically, emotionally, and socially which manifest as early and late premenstrual symptoms [28-30]. Some significant early symptoms include depression and anxiety, insomnia, hot flashes, or changes in sexual desires [29,30]. SJW (Hypericum perforatum) has an antidepressant mechanism in which the uptake of serotonin (5-HT), dopamine, and norepinephrine from the synaptic cleft is decreased or delayed [29-32].

Eatemadnia et al. conducted a double-blinded randomized controlled trial to assess the effects of SJW in postmenopausal depression and other premenstrual symptoms among 80 middle-aged women; 80% of women in the SJW intervention group did not have any depression; however, only 5.7% of no-depression individuals were found in the control group (p < 0.001). Depression intensity was also markedly reduced in the intervention group [29]. Grube et al. conducted a clinical trial in middle-aged women, in which 111 postmenopausal women were treated with SJW for 12 weeks duration, and results were found to be in favor of marked improvement of SJW in women's psychological and psychomotor symptoms [30,33,34].

SJW treatment - time of improvement

Several mechanisms of action are proposed for SJW [35]. Owing to its several actions, the time of this compound's effect on the treatment of depression can only be speculated. The primary mechanism cited for its antidepressant qualities is the inhibition of the reuptake of serotonin [31]. Data pointing to the long-term antidepressant effects of SJW is limited [36]. It has also been known to inhibit monoamine oxidase activity, translating into increased levels of norepinephrine [25]. In addition, SJW has gamma-aminobutyric acid (GABA) binding activity, which reduces GABA binding, resulting in lower levels of CNS depression [5]. Finally, it has shown promise in safety and effectiveness in cases where it has been used as the only treatment regimen. SJW has not yet received permission from the FDA due to its safety concern, although it is essential to note that drugs with similar actions and side effect profiles as SJW has been long approved by the FDA [6].

SJW treatment - most common adverse events registered

The most widely known adverse effect is a fatal increase in serotonin while prescribing serotonergic agents, possibly causing serotonin syndrome when coupled with certain antidepressants and monoamine oxidase (MAO)-I [16,35,37,38]. Serotonin syndrome is known to cause tachycardia, hypertension, mydriasis, and diaphoresis. Hyperthermia has also been recorded.

SJW is associated with various adverse effects involving the neurological system and the eyes, ears, liver, renal, and reproductive organs. Less commonly, SJW has been associated with hypertensive crisis, and inducing mania; adverse event reporting in identified trials was frequently general and focused on gastrointestinal discomfort and tolerability [6,17,35].

Adverse effects like allergic reactions, photosensitivity, nausea, headaches, diarrhea, skin irritation, and worsening psychotic symptoms in schizophrenia or bipolar disorder patients have also been documented. Anticoagulants and immunosuppressants are less effective when combined with SJW than traditional regimens. It has also been shown to make birth control pills less effective, making undesired conception possible [35,37].

While prescribing SJW, It is imperative to keep in mind that it is an alternative to antidepressants. Hence, care should be taken to avoid drug-drug interactions, especially with drugs utilizing the CYP 450 system (HIV protease inhibitors, CYP3A4 HIV non-nucleoside reverse transcriptase inhibitors, ciclosporin, tacrolimus, irinotecan, imatinib mesylate, digoxin, oxycodone, and warfarin) [6,35,37,38]. There has also been one report of severe sedation when SJW was taken in conjunction with paroxetine in an elderly patient [18]. However, these adverse effects are fewer when compared to the traditional modalities used [16,37,38].

## Conclusions

We sought to identify the advantages and disadvantages of using SJW to treat depression. First, it is essential to acknowledge that SJW has been used as a treatment measure in Germany. Despite being only listed as a dietary supplement by the FDA and not a drug, SJW has shown to be comparable if not more efficacious than most standard treatment options for depression. Some randomized controlled trials go so far as to say that SJW is a better option than even fluoxetine due to its better tolerability and safety profile. SJW has also been studied as a possible avenue for treating post-menopausal depression and some post-menopausal symptoms such as hot flashes.

Nevertheless, we cannot conclude that SJW is the appropriate alternative to tricyclic antidepressants or SSRIs. When studied as a combination regimen with tricyclic antidepressants or SSRIs, data suggests no incremental change in efficacy when treating depression. Given that its mechanism of action does rely on serotonin re-uptake, serotonin syndrome is a common adverse effect discussed. Studies have also shown that side effects such as headaches, nausea, and photosensitivity can deter patients from completing the course of treatment. Despite all of this, SJW does prove to be an exciting piece of pharmacotherapy in the realm of mental health. More prospective studies will help us better understand its efficacy in mild and moderate depression and its ability to serve as a long-term agent. Considering its mechanism of action, its role in relieving patients suffering from an anxiety disorder is also worth considering.
